# Strain-Dependent Differences in Inflammatory/Immune Activity in Cutaneous Wound Tissue Repair in Rats: The Significance of Body Mass/Proneness to Obesity

**DOI:** 10.1155/mi/5525557

**Published:** 2025-03-26

**Authors:** Jelena B. Kulas, Aleksandra D. Popov Aleksandrov, Dusanka D. Popovic, Anastasija Lj. Malesevic, Maja M. Cakic Milosevic, Milena V. Kataranovski, Ivana I. Mirkov, Dina M. Tucovic

**Affiliations:** ^1^Immunotoxicology Group, Department of Ecology, Institute for Biological Research “Sinisa Stankovic” – National Institute of the Republic of Serbia, University of Belgrade, Belgrade, Serbia; ^2^Institute of Zoology, Faculty of Biology, University of Belgrade, Belgrade, Serbia

**Keywords:** cutaneous wound healing, immune/inflammatory activity, obesity proneness, rat strains

## Abstract

Inflammatory/immune cells and mediators are substantial for wound healing because they orchestrate biological activities in this complex process. Among factors that affect wound healing, obesity, and metabolic diseases are among the most significant, particularly because of a relationship between obesity and a prediabetic state with immune reactivity. Using Dark Agouti (DA) and Albino Oxford (AO) rats, which differ in immune responses as well as in proneness to obesity, we examined the impact of these intrinsic factors on cutaneous wound healing. Dynamics of the process were monitored at days 3, 5, and 7 post-wounding parallel in both rat strains by analysis of selected basic aspects of the wound repair process (cytokine and growth factor responses) in granulation tissue. Strain-related differences in the extent of reduction of the wound area were shown, which coincided with differential proinflammatory and immune-regulatory cytokines, as well as growth factors response in these rats. Some of these differences seem related to their dissimilarities in the proneness to obesity. Results in this study extended so far known differences in inflammatory/immune responses to a variety of stimuli between AO and DA rats and showed, for the first time, immune-based differences in wound healing between rats that differ in body mass (BM) and obesity proneness (under ad libitum feeding conditions with normal rodent chow).

## 1. Introduction

As the body`s integumentary system [1, 2], the skin is exposed to a harsh environment, often with a violation of integrity. Disturbance of the epidermis or dermis initializes wound healing, a complex process involving mutually related and overlapping biological activities, which include cell migration and proliferation, production of extracellular matrix (ECM), growth factors, and cytokines. It is accomplished through several overlapping stages of inflammation, proliferation, and tissue remodeling [3–7]. Immune system cells and mediators largely mediate these phases via the orchestration of biological activities in this complex process [8].

The inflammatory phase starts with hemostasis (characterized by platelet aggregation and coagulation cascade activation with fibrin mesh formation), which is followed by inflammatory and other cell recruitment [9]. Infiltrated polymorphonuclear (PMN) cells, predominantly neutrophilic leukocytes, fight against infection, perform debridement of necrotic tissue, and produce soluble factors, which attract/activate fibroblasts, keratinocytes and endothelial cells [9]. The initial rapid influx of PMNs is followed by an entry of monocytes, which partly differentiate into macrophages (Mphs) within the wound. With the progression of the inflammatory phase, the wound Mph phenotype changes, acquiring a spectrum of phenotypes, which enable both inflammation as well as tissue repair promotion and transition to the proliferative phase [10]. Production of proinflammatory cytokines IL-1*β*, tumor necrosis factor (TNF), and IL-6, reactive oxygen species (ROS), and nitric oxide (NO) promotes host defense and damaged tissue removal, and mediators such as interleukin-4 (IL-4) and interleukin-10 (IL-10) as well as arginase are required for tissue repair and resolution of inflammation [11, 12].

The creation of granulation tissue represents a transition to a phase of tissue repair (proliferative phase). Fibroblasts, keratinocytes, noninflammatory Mphs, and endothelial cells produce a variety of cytokines and growth factors, including transforming growth factor *β*1 (TGF-*β*1) [9] and vascular endothelial growth factor (VEGF), which drive ECM deposition, wound closure and angiogenesis/restructuring of the wound vascular system [13, 14]. Deposition of collagens (first collagen III and later type I), mostly by fibroblasts, contributes to replacing the provisional fibrin-rich matrix with a more substantial granulation tissue, which serves as a matrix for activities in the proliferation phase. The remodeling or maturation phase is the end phase based on the regression of neovasculature, ECM reorganization, and reconstitution of granulation tissue to a scar.

Many factors can affect cutaneous wound healing and are classified as local, directly influencing the wound itself, and systemic, not directly related to a wound location [15, 16]. Systemic factors include intrinsic physiological factors (such as age and gender), internal pathophysiological factors (including certain disease states and immune-compromised conditions), and external factors (medication, alcohol, smoking, and nutrition). Obesity and metabolic diseases, such as diabetes, are among the most significant internal pathophysiological factors of wound healing impairment [17, 18]. The use of different rodent (mouse and rats) strains showed that the extent of wound healing impairment varies between murine diabetic strains [19] and that the effect of diabetes state on wound healing differs between several strains [20]. Some of the mouse strains in these studies are immune-biased [21, 22]. Moreover, a delay in cutaneous wound healing in high-fat diet-induced obese mice and rats [23, 24] was associated with changes in some inflammatory/immune parameters in the wounds. This, along with data showing immunity as a link between obesity and prediabetic state [25], raises the question of the significance of immune reactivity to strain-related differences in the effects of wound healing-disturbing factors. However, to the best of our knowledge, there are no investigations devoted specifically to the examination of the impact of these factors on cutaneous wound healing.

Dark Agouti (DA) and Albino Oxford (AO) rats differ in immune reactivity both locally (in the skin) [26, 27] and in systemic immune reactions [28–31]. These rats also differ in body size and fatness under ad libitum feeding conditions with normal rodent chow, with DA rats presented as obesity-resistant rats [32]. Although there are studies of differences in body size/adiposity in common rat strains [33] and their obesity phenotypes [34], there are no data concerning immunity in these rat strains. AO and DA rats were used in the present work for the examination of inflammatory/immune activity in cutaneous wound healing. Selected basic aspects of the wound repair process were analyzed, including cytokine and growth factor responses in granulation tissue. Data to be shown demonstrated differences in the extent of reduction of wound area that coincided with differential proinflammatory and immunoregulatory cytokine as well as growth factor response in these rats. Some of them seem related to differences in the proneness to obesity. Results in this study extended so far known differences in inflammatory/immune responses to a variety of stimuli between two rat strains and showed, for the first time, immune-based differences in wound healing between rats, which differ in body mass (BM) and obesity proneness (under ad libitum feeding conditions with normal rodent chow).

## 2. Materials and Methods

### 2.1. Animals

Animal treatment and experimental procedures were carried out in compliance with Directive 2010/63/EU on the protection of animals used for experimental and other scientific purposes and were approved by the Veterinary Directorate, Ministry of Agriculture, Forestry and Water Management (No. 000491937 2023 14841 002 000 323 022). Male AO and DA rats, 10–12 weeks old, were conventionally housed (12-h light/dark cycle, ambient temperature of 22 ± 2°C and 60% relative humidity) at the animal facility of the Institute for Biological Research “Sinisa Stankovic,” Belgrade, Serbia). They have had unlimited access to standard rodent pelleted food (consisting of 3.2% fat, 49.3% carbohydrates, 20.3% proteins, and 12.9% fibers; energetic value 1405 kJ/100 g) and water. Food intake was determined in animals kept 24 h in metabolic cages (following 3-day adaptation).

### 2.2. Animal Morphometric Characteristics

BM was determined using a precise digital electronic scale with a capacity of 3 kg and a sensitivity of 0.01 g. Naso-anal body length was measured in animals under inhalation anesthesia (Vetflurane, Virbac, France, 3% minimal alveolar concentration) using a nonextensible thread, and readings were taken using a ruler with a sensitivity of 0.1 cm. Obtained values for BM (expressed in g) and length (expressed in cm) were used for calculating BM index (BMI) (by dividing BM by square length) and Lee index (dividing cubic root of weight by naso-anal length). Body surface area was calculated using the following formula: area (in cm^2^) = 9.6 × BM^2/3^.

### 2.3. Fasting Blood Glucose

Fasting blood glucose was determined in blood obtained from the tail tip using a glucometer (Sensimac, IMACO GmbH, Lüdersdorf, Germany) following 4 h fast.

### 2.4. Wounding and Macroscopic Analysis

For the assessment of wound healing, animals of each strain were randomly divided into three groups (*n* = 9 animals per group). Wounding of animals and following procedures were as previously described [23]. Two full-thickness excisional wounds (1 cm in diameter) were performed on the shaved back of anesthetized animals. The wound was not sutured or covered. Wound closure was evaluated daily by tracing wound margins using a plastic transparent sheet placed over the wound. The wound area was calculated from digitalized transparent sheets using ImageJ software, and changes were expressed as a percentage of the initial wound areas. On days 3, 5, and 7 after the infliction of the wound, one group of animals of each strain was sacrificed, and wound tissue with a thin margin of uninjured skin was collected for histological and functional analysis.

### 2.5. Clinical Hematological Parameters

On days 1 and 3 following skin excision, blood was withdrawn from the abdominal artery to assess systemic inflammatory response in wounded animals [35]. As a control, animals without wounds were used. Hematological parameters were determined automatically by Siemens ADVIA 120 flow cytometer (Terytown, New York, USA) using commercially available reagents.

### 2.6. Histological Examination

On days 3, 5, and 7 after the infliction of the wound, wound tissue with a thin margin of uninjured skin was collected for histological and functional analysis. The samples of wounds were fixed in 4% formaldehyde and routinely processed for light microscopy. Five μm thick tissue sections stained with hematoxylin and eosin (HE) were used for histological evaluation of events related to wound healing. Selected characteristics of wound tissue were graded semi-quantitatively based on an arbitrary scoring scale ranging from 0 to 3 (0—no evidence, 1—scant, 2—moderate, and 3—prominent), modified from McMinn [36] and de Moura Estevão et al. [37]. Two nonadjacent tissue sections per animal were analyzed and photographed using a Leica DMLB light microscope (Leica Microsystems, Wetzlar, Germany) equipped with a Leica DFC295 camera and LAS Core software.

### 2.7. Isolation of RNA, Reverse Transcription, and Real-Time Polymerase Chain Reaction (RT-PCR)

Collected granulation tissue samples were immediately homogenized in mi-Total RNA Isolation Kit (Metabion, Martinsried, Germany) according to the manufacturer's recommendation. Isolated RNA (1 µg) was reversely transcribed using random hexamer primers and Moloney murine leukemia virus (MMLV) reverse transcriptase (Fermentas, Vilnius, Lithuania), following manufacturer's instructions. Obtained complementary DNAs (cDNAs) were amplified using Power SYBR Green PCR Master Mix (Applied Biosystems, Foster City, California, USA) based on the recommendations of the manufacturer in a total volume of 20 μl in an ABI PRISM 7000 Sequence Detection System (Applied Biosystems). Thermocycler conditions consisted of an initial step at 50°C for 5 min, followed by a step at 95°C for 10 min and a subsequent 2-step PCR program at 95°C for 15 s and 60°C for 60 s for 40 cycles. PCR primers (forward/reverse) used in the study were *β*-actin—5′−CCCTGGCTCCTAGCACCAT-3′/5′-GAGCCACCAATCCACACAGA-3′; IL-1*β—*5′-CACCTCTCAAGCAGAGCA-3′/5′-GGGTTCCATGGTGAAGTCAAC-3′; IL-6—5′-CCCTTCAGGAACAGCTATGA-3′/5′-TGTCAACAACATCAGTCCCAAG-3′; TNF—5′-TCGAGTGACAAGCCCGTAGC-3′/5′-CTCAGCCACTCCAGCTGCTC-3′; TGF-*β*1—5′-CCCTGCCCCTACATTTGGA-3′/5′-ACGGTGATGCGGAAGCAC-3′; IL-10—5′-GAAGACCCTCTGGATACAGCTGC-3′/5′- TGCTCCACTGCCTGGCTTTT-3′; inducible nitric oxide synthase (iNOS)—5′-TTCCCATCGCTCCGCTG-3′/5′-CCGGAGCTGTAGCACTGCA-3′; arginase 1 (Arg1)—5′-TGGACCCTGGGGAACACTAT-3′/5′- GTAGCCGGGGTGAATACTGG-3′; VEGF—5′-GGGCCTCTGAAACCATGAACT-3′/5′-ACGTCCATGAACTTCACCACTTC-3′; Col3a1—5′-CTGGTCCTGTTGGTCCATCT-3′/5′-ATGCCATTAGAGCCACGTTC-3′; Col1a1—5′-ACATGCCGTGACCTCAAGAT-3′/5′-ATGTCCATTCCGAATTCCTG-3′. PCR results were analyzed with 7500 System Software (Applied Biosystems) and calculated as 2^−dCt^, where dCt was the difference between threshold cycle (Ct) values of the specific gene and the endogenous control (*β*-actin).

### 2.8. Sponge Preparation, Implantation, and Collection of Fluid and Cells

To investigate cell migration during the wound healing process, we employed a subcutaneously implanted polyvinyl sponge model, which is often used in the investigation of a variety of aspects of wound healing [38]. Sterile (by autoclaving 20 min at 121°C) polyvinyl sponges (1 × 1 × 3.5 cm) were aseptically implanted subcutaneously through incisions made on either side of the dorsal mid-line of rats previously clipped of hair and wiped with 70% ethanol. Following sponge implantation, animals were housed individually.

After 18 h, rats were anesthetized, and sponges were quickly taken through skin incisions. Sponges from each rat were gently squeezed into centrifuge tubes. Wound fluid was stored at −20°C for cytokine detection. The cell pellet was resuspended in a culture medium for further evaluation.

### 2.9. Leukocyte Counts and Flow Cytometry

Cells isolated from sponges were counted by the improved Neubauer hemacytometer. Flow cytometric analysis was used for the determination of granulocytes (FITC labeled mouse anti-rat HIS48, ThermoFisher Scientific, Massachusetts, USA) and monocytes/Mphs (Alexa Fluor 488 labeled mouse anti-rat CD68, AbD Serotec, Serotec Ltd., Oxford, UK). Proportion of granulocytes was determined following incubation of cells (1 × 10^6^) on ice for 30 min with HIS48, washing twice with PBS, and fixation with 1% paraformaldehyde. For detection of CD68^+^-cells, sponge cells (1 × 10^6^) were fixed in 1% paraformaldehyde (Serva, Heidelberg, Germany) in PBS, permeabilized for 15 min with PBS containing 0.2% Tween-20 (Sigma Chemical Co., St. Louis, MO, USA) and then incubated for 30 min with mouse anti-rat CD68-Alexa Fluor 488.

For measurement of intracellular ROS by flow cytometry dihydrorhodamine 123 (DHR 123) assay based on the oxidation of DHR 123 to fluorescent rhodamine 123 by hydrogen peroxide was used [39]. Cells (1 × 10^6^) were incubated for 1 h in the medium in the presence of 4 μM DHR 123 (Life Technologies Corp., Carlsbad, CA, USA). Following incubation, the cells were washed with PBS and analyzed by flow cytometry. Fluorescence intensity was measured on a CyFlow Space flow cytometer (Partec, Munster, Germany). Cells were gated according to side and forward scatter, and a minimum of 10,000 events/samples were acquired each time and analyzed using FlowMax software (Partec).

### 2.10. Myeloperoxidase (MPO)

MPO activity of the cells was assessed on the basis of the oxidation of o-dianisidine dihydrochloride [40]. Briefly, 33 μl of cell lysate was added to 966 μl of substrate solution (0.167 mg/ml o-dianisidine dihydrochloride and 0.0005% H_2_O_2_ in 50 mM potassium phosphate buffer, pH 6.0). Absorbance was read at 450 nm at 3 min intervals up to 10 min against the standard of MPO.

### 2.11. Enzyme-Linked Immunosorbent Assays (ELISAs)

Cytokine concentrations were determined in wound fluid and supernatant of cells (5 × 10^5^ cells/well of 96-well plate) cultured for 48 h in the medium. Concentrations of IL-1*β*, IL-6, TNF (R&D Systems, Minneapolis, USA), and IL-17 (ThermoFisher Scientific, Massachusetts, USA) were determined using commercially available ELISA sets according to the manufacturer's instructions. The standard curve generated using known amounts of the respective set provided recombinant cytokines was used to calculate cytokine titers.

### 2.12. Statistical Analysis

The results were pooled from two independent experiments with four or five animals per group per experiment (a total of nine animals per group) and presented as mean ± standard deviation. For the comparison of histological scores, the Kruskal–Wallis test was used, while other data were compared by analysis of variance (one-way ANOVA) followed by Tukey's test (STATISTICA 7.0, StatSoft Inc, Tulsa, OK) or by Student's *t*-test. *p*-Values < 0.05 were considered significant.

## 3. Results

### 3.1. Animal Morphometric Characteristics

BM is higher in AO compared to DA rats (Table 1). Higher values of BMI and the Lee obesity index were observed in AO rats, with values over 0.68 (for BMI) and 300 (Lee index) that are defined as threshold values for obesity [41, 42].

Despite differences in BM, there was no significant difference in the ratio of wound area to the body surface (0.14% ± 0.03% in DA, 0.12% ± 0.03% in AO, *p*=0.1).

### 3.2. Fasting Blood Glucose

As diabetes was shown to affect wound healing [19, 20], we measured fasting blood glucose next. In 12-week-old DA and AO rats, no differences were noted in glucose level (5.9 ± 0.3 mmol/l in DA vs. 6.1 ± 0.3 mmol/l in AO rats).

### 3.3. Peripheral Blood Leukocyte (PBL) Response to Skin Excision

Following skin excision, no changes were noted in the number of total white blood cells (Table 2), but an increase in relative neutrophil numbers, followed by a decrease in lymphocytes, was noted in wounded DA rats on day 1 post-wounding. In contrast, a decreased relative number of neutrophils and increased lymphocytes were noted in AO rats.

### 3.4. Dynamics of Wound Closure

**Figure 1:**
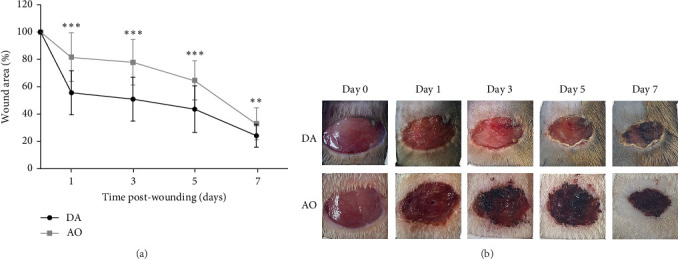
Wound closure dynamic in DA and AO rats. (A) Wound closure is expressed as a percentage of the initial wound areas. (B) Representative photographs of wounds. Results are presented as mean ± S.D. for a total of nine animals per time point from two independent experiments. Data are analyzed using ANOVA with Tukey's multiple comparisons test. Statistically significant at *⁣*^*∗∗*^*p* < 0.01 and *⁣*^*∗∗∗*^*p* < 0.001 for DA vs. AO rats. ANOVA, analysis of variance; AO, Albino Oxford; DA, Dark Agouti.

A progressive decrease in wound area was noted in both strains, but wound areas were larger in AO as compared to DA rats during the first 7 days post-wounding (Figure 1A). Thereafter, the wound area equalized.

### 3.5. Wound Histology

**Figure 2:**
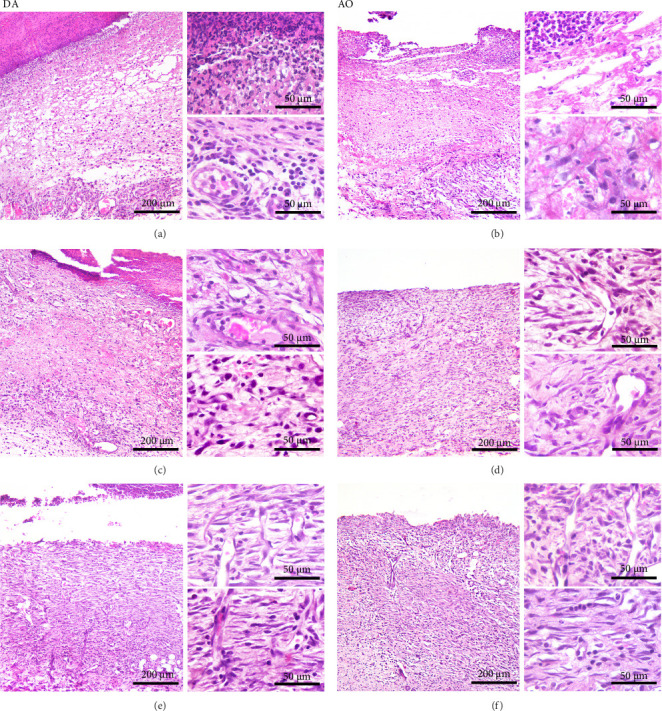
Histological analysis of wound tissue. Representative photomicrographs of HE-stained full-thickness wounded skin in DA and AO rats at day 3 (A and B), 5 (C and D), and 7 (E and F) post-wounding. Representative photomicrographs in low-power field, scale bar = 200 μm. Insets: representative photomicrographs in the high-power field, scale bar = 50 μm. AO, Albino Oxford; DA, Dark Agouti; HE, hematoxylin and eosin.

Histological analysis showed wound healing activities, that is, inflammation and proliferation in both strains (Figure 2). On day 3 (Figure 2A,B), the wounds were in the late inflammatory phase with inflammatory infiltrate consisting of PMN and mononuclear (MN) cells. Most PMNs visible on tissue sections were located in the wound tissue facing the scab (Figure 2A,B, upper inset). Inflammatory infiltration and especially PMN presence were more intense in DA rats (PMN score 2.7 ± 0.6 in DA vs. 1.7 ± 0.6 in AO rats, *p*=0.028). Dilated blood vessels with activated endothelium were seen at the bottom of the wound in both groups of animals, and some of them were congested in the DA rats. The perivascular spaces were occupied by enlarged stellate fibroblasts in the AO rats (Figure 2B, lower inset), whereas MNs predominated in the DA animals (Figure 2A, lower inset). The remaining portion of the wound bed was filled with a provisional matrix. Semiquantitative analysis showed either lack or weakly expressed edema (0.7 ± 0.3) in AO as compared to DA rats (2.0 ± 0.8, *p*=0.039).

The main histological events on day 5 of wound healing (fibroplasia, deposition of ECM, and neovascularization) were clearly visible in both rat strains (Figure 2C,D). Restoration of the vascular network by angiogenesis was evidenced by many dilated, circular, tubular, or irregularly shaped blood vessels (Figure 2C, upper inset; 2D, insets). The edema decreased in both groups compared to day 3 but remained more pronounced in the DA animals (edema intensity 1.5 ± 0.5 and 1.3 ± 0.6 on days 5 and 7 post-wounding). The granulation matrix produced by synthetic active fibroblasts appeared to be slightly more mature in the AO animals, as evidenced by a more pronounced tendency of the fibroblasts to arrange themselves in an orderly fashion and parallel to the wound surface (Figure 2C,D, insets). Moderate to pronounced MN and, to a lesser extent, PMN cell infiltration of the wound tissue and partial reepithelialization were observed in all animals. Reepithelialization was evident at this time point in both rat strains and was similar in DA and AO rats.

On day 7 after injury, the granulation tissue was well organized, and spindle-shaped fibroblasts became the predominant cell type in the wound area (Figure 2E,F). The fibroblasts were arranged parallel to the wound surface (Figure 2E, insets; 2F, lower inset) except at the upper portion of the wound in the AO rats (Figure 2F, upper inset). MN cell infiltration was markedly reduced, while PMNs were sparse or almost completely absent. Blood vessels were visibly more numerous in DA rats. Their lumen was reduced and often tubular. Many of the tubular blood vessels were oriented perpendicular to the wound surface (Figure 2E, insets; 2F, upper inset), but in the lower part of the wound bed of the AO rats (Figure 2F, lower inset), they were more frequently arranged horizontally. Overall, the wound vasculature appeared more intense in the DA rats. The epidermis covered a substantial part of the wound but was still immature, and the degree of reepithelialization was similar in both rat strains.

### 3.6. Oxidative Activity in Granulation Tissue

**Figure 3:**
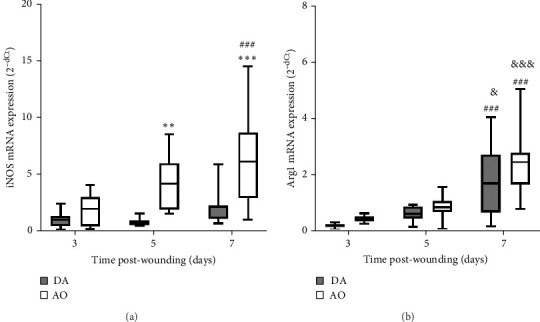
Oxidative activity in granulation tissue in DA and AO rats. (A) iNOS mRNA expression. (B) Arginase 1 (Arg1) mRNA expression. Data are presented as mean ± S.D. for a total of nine animals per time point (from two independent experiments) and compared using ANOVA with Tukey's multiple comparisons test. Statistically significant at *⁣*^*∗∗*^*p* <  0.01 and *⁣*^*∗∗∗*^*p* <  0.001 for DA vs. AO rats; ^###^*p* < 0.001 for comparison with day 3 within the strain; and ^&^*p* < 0.05 and ^&&&^*p* < 0.001 for comparison with day 5 within the strain. ANOVA, analysis of variance; AO, Albino Oxford; DA, Dark Agouti; iNOS, inducible nitric oxide synthase; mRNA, messenger ribonucleic acid.

During the examined period, iNOS messenger ribonucleic acid (mRNA) was noted in the granulation tissue of individuals of both strains, but with higher levels in AO compared to DA at days 5 and 7 (Figure 3A). The highest levels of arginase mRNA were detected in both strains at day 7 post-wounding, with levels that are not different between strains (Figure 3B).

### 3.7. Cytokine and Growth Factor Response in Granulation Tissue

**Figure 4:**
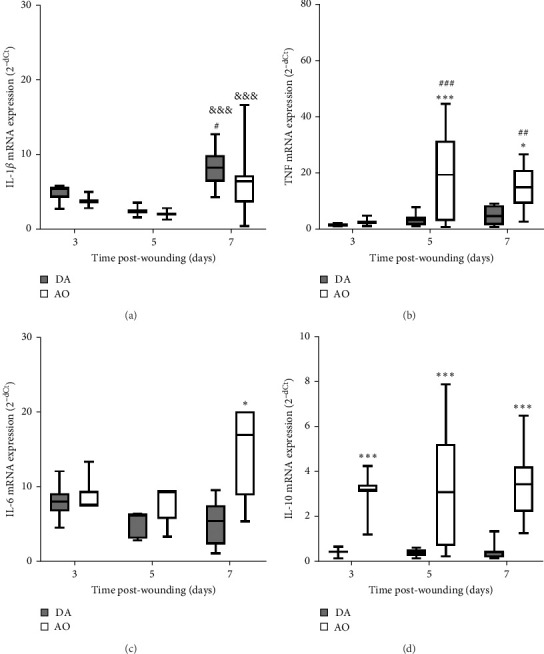
Cytokine response in granulation tissue in DA and AO rats. (A) IL-1*β* mRNA expression. (B) TNF mRNA expression. (C) IL-6 mRNA expression. (D) IL-10 mRNA expression. Data are presented as mean ± S.D. for a total of nine animals per time point (from two independent experiments) and compared using ANOVA with Tukey's multiple comparisons test. Statistically significant at *⁣*^*∗*^*p* < 0.05 and *⁣*^*∗∗∗*^*p* < 0.001 for DA vs. AO rats; ^#^*p* < 0.05, ^##^*p* < 0.01, and ^###^*p* < 0.001 for comparison with day 3 within the strain; and ^&&&^*p* < 0.001 for comparison with day 5 within the strain. ANOVA, analysis of variance; AO, Albino Oxford; DA, Dark Agouti; IL-10, interleukin-10; mRNA, messenger ribonucleic acid; TNF, tumor necrosis factor.

The presence of IL-1*β* was noted during the entire period, with up-growth at day 7 in both strains (Figure 4A). Increased TNF mRNA levels were observed in AO rats at days 5 and 7 and are higher compared to DA rats (Figure 4B). IL-6 expression stayed unchanged during the entire period, with significantly higher values in AO as compared to DA rats at day 7 following wounding (Figure 4C). Measurements of IL-10 revealed higher values of this cytokine (Figure 4D) during the examined period in granulation tissue in AO rats.

**Figure 5:**
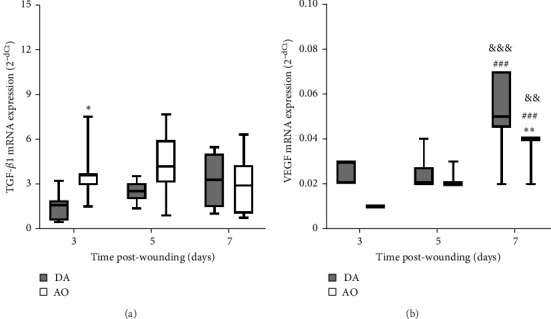
TGF-*β*1 (A) and VEGF (B) mRNA expression in granulation tissue in DA and AO rats. Data are presented as mean ± S.D. for a total of nine animals per time point (from two independent experiments) and compared using ANOVA with Tukey's multiple comparisons test. Statistically significant at *⁣*^*∗*^*p* < 0.05 and *⁣*^*∗∗*^*p* < 0.01 for DA vs. AO rats; ^###^*p* < 0.001 for comparison with day 3 within the strain; and ^&&^*p* < 0.01 and ^&&&^*p* < 0.001 for comparison with day 5 within the strain. ANOVA, analysis of variance; AO, Albino Oxford; DA, Dark Agouti; mRNA, messenger ribonucleic acid; TGF-β1, transforming growth factor β1; VEGF, vascular endothelial growth factor.

Examination of TGF-*β*1 showed the presence of TGF-*β*1 mRNA in granulation tissue of both strains with significantly higher values at day 3 in AO rats (Figure 5A). In both rat strains, VEGF mRNA levels increased 7 days following wounding, but VEGF enhancement was less in AO rats (Figure 5B).

### 3.8. Collagen Synthesis

**Figure 6:**
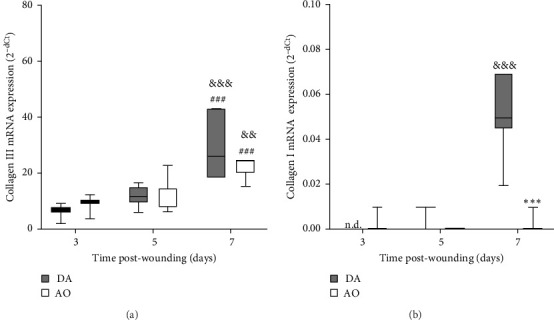
Collagen synthesis in granulation tissue in DA and AO rats. (A) Collagen III mRNA. (B) Collagen I mRNA expression. Data are presented as mean ± S.D. for a total of nine animals per time point (from two independent experiments) and compared using ANOVA with Tukey's multiple comparisons test. Statistically significant at: *⁣*^*∗∗∗*^*p* < 0.001 for DA vs. AO rats; ^###^*p* < 0.001 for comparison with day 3 within the strain; and ^&&^*p* < 0.01 and ^&&&^*p* < 0.001 for comparison with day 5 within the strain. ANOVA, analysis of variance; AO, Albino Oxford; DA, Dark Agouti; mRNA, messenger ribonucleic acid; n.d., not detectable.

The highest levels of collagen III mRNA were observed in both strains at day 7 following wounding (Figure 6A). A pronounced increase of collagen I at day 7 was seen in DA rats solely (Figure 6B).

### 3.9. Migration of Inflammatory Cells to the Wound

As results obtained in the excision wound model suggest differences in cell infiltrations in DA and AO rats, we employed another model to evaluate these differences. A similar number of cells migrating to the wound at day 1 following wounding was noted in these two strains (Table 3), but a higher proportion of neutrophils (HIS48^+^ cells) and a lower proportion of monocytes/Mphs (CD68^+^ cells) were detected in AO compared to DA rats. Examination of activities of cells recovered from the incision wound on day 1 revealed lower MPO activity in AO compared to DA rats. Nevertheless, higher production of ROS, determined by relative expression level (MFI) of DHR 123, was also observed in AO relative to DA rats, despite similar percentages of DHR^+^ cells in these rats, demonstrating strain differences in oxidative burst capacity between DA and AO rats. Additionally, infiltrated cells isolated from AO rats on day 1 following incision were shown to produce a higher amount of IL-1*β* but less IL-6 and IL-17 than cells isolated from DA rats (Table 3). Migration of cells occurred in a wound environment characterized by lower levels of IL-1*β*, IL-6, and IL-17 but higher levels of TNF in AO rats compared to DA.

## 4. Discussion

In this study, immune-related activity in granulation tissue of wounded skin was analyzed in DA and AO rats, shown previously to differ in immune responsiveness to various insults and which differ in body type. The presence of inflammatory activity was noted in both strains, but some aspects of inflammation, including expression of genes for iNOS, TNF, and IL-6, were more pronounced in AO rats. Greater expression of immunoregulatory IL-10 and wound-healing promoting factor TGF-*β*1, but a lower expression of VEGF and collagen I was also observed in AO rats.

Despite having similar caloric intake/food consumption, AO and DA rats differ in BM. Judging by morphometric parameter values, including BMI, one of the most objective methods to determine the presence/extent of obesity [18], and Lee's index, which positively correlates with retroperitoneal fat [43], AO rats may be considered obesity-prone. It is interesting to note that the composition of gut bacterial microbiota differs between DA and AO rats [44], which can also contribute to obesity tendencies [45]. Similar glucose levels noted in 12-week-old DA and AO rats exclude the potential impact of diabetes on wound healing, as this state is also known to affect wound healing [19, 20]. Based on these findings, it might be assumed that a differential rate of wound area reduction in AO and DA rats might be ascribed to differences in their body type/adiposity, as it is known that overweight and obesity delay cutaneous wound healing [17, 18].

The difference in the wound area reduction rate between DA and AO rats is in broad line with data that demonstrated the contribution of mouse strain to wound healing impairment by external [46, 47] or internal factors [20].

The greater presence of PMN early in the course of granulation tissue formation in DA rats is a consequence of a more intense infiltration, as these cells are rare in intact skin and are the first cells to appear in wounded skin [48]. Indeed, on day 1 following incision wounding, a greater proportion of neutrophils has been observed in subcutaneously implanted polyvinyl sponges inserted into DA than in those from AO rats, which corroborated greater MPO activity of cells infiltrating injured tissue of DA rats. It should also be noted that the proportions of PMN on day 3 following sponges placement remain higher in DA rats (23.7% ± 7.0%) as compared to those in AO rats (13.1% ± 3.6%, *p*=0.007) (I.M., unpublished). This is in line with data demonstrating a relationship between impaired neutrophil recruitment and impaired wound healing [49]. Neutrophil influx reflects absolute requirements for these cells at the site of injury and the rise of their numbers in circulation could contribute to their migration. Differences in PMN migration between these two rat strains were also noted in other experimental settings. An extended influx of neutrophils following intraperitoneal injection of *E. coli* was previously observed in DA compared to AO rats [50], and more effective neutrophil clearance was observed in rats of AO strain during peritonitis [51]. Besides lower neutrophil infiltration in obesity-prone rat strains, the activity of these cells might as well be impaired in AO rats. In this context, our previous investigation of pulmonary immune response to fungal infection in DA and AO rats revealed lower intracellular MPO activity in AO rats [30]. Other PMN functions, such as the formation of neutrophil extracellular traps, are also significantly impaired in obese animals [52]. Migration of cells in DA and AO rats occurred in an environment that is different in cytokine content. In DA rats, higher levels of IL-1*β* and IL-17 were noted and might contribute to the robust influx of neutrophils into the wounds [53, 54]. Interestingly, wound cells observed on day 1 following incision in DA rats produced higher levels of inflammatory IL-6 and IL-17 but lower IL-1*β* when compared to wound cells from AO rats, indicating that other cells besides infiltrating leukocytes might contribute to inflammatory milieu in the wounds. In this context, it is known that keratinocytes and mast cells are readily available sources of cytokines capable of storing and releasing the presynthesized IL-1 [53] and TNF [55], respectively, and that IL-17 is mainly produced by resident skin cells [54, 56].

Resident and infiltrating monocytes and Mphs may progress from one functional phenotype to another in response to changes in their tissue environment [57], and it might be assumed that complex wound environment in AO rats was permissive for cell inflammatory M1-like polarization, not for sequential phenotype changes toward anti-inflammatory M2 milieu that favor the wound closure [58].

The more prominent presence of monocytes and Mphs in the incision wound of AO rats compared to DA rats probably also mediated the more intense cellular oxidative response measured by the DHR test, which can also further perpetuate Mph inflammatory polarization. Moderate elevation of ROS in the early stages of wound healing facilitates the reestablishment of a sterile ecological niche at the wound site and activation of Mphs and their metabolic reprograming toward glycolysis. When produced in excess or in the later phases, ROS can be deleterious [59]. In this context, our preliminary results showed a higher proportion of cells demonstrating oxidative burst (22.0% ± 7.4% DHR^+^ cells in DA vs. 48.5 ± 10.5 in AO, *p*=0.003) and a higher capacity to produce ROS (6.8 ± 0.5 MFI vs. 9.6 ± 1.4, *p*=0.003, in DA and AO rats, respectively) in AO rats at day 3 in incision wounds, suggesting prolonged ROS presence in wounds of this strain. Excessive ROS-producing capacity was associated with obesity and metabolic syndrome [60], and ROS scavengers were efficiently used in treatments of chronic diabetic wounds [61]. Likewise, Mphs from AO rats were more efficient in secreting reactive oxygen products than Mphs from DA rats, and this was observed during phagocytosis-induced as well as during phagocytosis-independent activation of protein kinase C [51].

An increase in iNOS mRNA expression in granulation tissue in AO rats most probably represents an attempt of wound healing improvement, as its product NO is an important and well-established mediator of this process [62, 63].

A time-dependent increase in arginase mRNA in both strains points out the importance of this enzyme for the physiological progression of wound healing in both strains, as the reduction of arginase is a feature of delayed healing in mice and humans [64]. However, the concomitant increase of both iNOS and arginase in AO rats indicates some impairment in tissue repair, as a temporal difference in the expression of these two enzymes is characteristic of the physiologic progression of cutaneous wound healing in rats [65]. As Mphs are the main source of both arginase and iNOS in mice [64] and rats [65], the association of delay in wound closure and increased accumulation of proinflammatory monocytes/Mphs in mice with obese phenotype induced by a high-fat diet [24], might be relevant for slower wound closure in obese AO rats. Nevertheless, even though the conversion of arginine to urea via the arginase pathway promotes collagen synthesis and tissue repair [66], increased Arg1 expression on day 7 in both rat strains was paralleled by the increase of collagen III expression, but not by the collagen I expression in wound tissue of AO rats. It may be worth noting here that a primary stimulator of type I collagen production, collagen chaperon heat shock protein 47 (HSP47) [67], was significantly less expressed in the joint tissue of AO relative to DA rats [68]. As lower HSP47 expression, by inappropriate collagen synthesis and less differentiation of fibroblasts into myofibroblasts, may abrogate wound healing in diabetic compared to control wounds [69, 70], it may also probably be implicated in altered rate of wound closure in the tissue of AO rats.

Although levels of IL-1*β* mRNA were increased in both strains, more pronounced mRNA expression of TNF and IL-6 in granulation tissue of AO rats indicates the persistence of inflammation. Prolonged inflammation is a known cause of poor wound healing [17]. High levels and extended presence of TNF inhibit wound reepithelization, and increased levels of IL-1*β*, IL-6, and TNF are found in chronic wounds [71]. Given the increased expression of TNF and IL-6 noted in mice with high-fat diet-driven obesity phenotype [24], it is assumable that the inflammation observed in AO rats might be related to their proneness to obesity. Interestingly, IL-17 shown to be lower in wound fluid at day 1 in AO rats when compared to DA, can not be detected in granulation tissue at later time points.

Immunoregulatory cytokine IL-10 facilitates the transition to the proliferative phase by modulating the type of cells that infiltrated the wound and by cytokine regulation [72], so an increase of IL-10 might be explained as a need to control this transit by inhibition of IL-1*β*, TNF, and IL-6 increase in granulation tissue of AO rats.

Higher expression of TGF-*β*1 mRNA early in the proliferative phase in AO rats might be viewed as an attempt at wound healing improvement. TGF-*β*1 functions as a wound-healing promoting factor, as it initiates granulation tissue formation (an essential feature of wound repair and the hallmark of an established healing response) by upregulating the expression of angiogenic growth factors (including the most potent VEGF) and through its involvement in collagen production (particularly types I and III) [10, 73, 74]. Higher TGF-*β*1 mRNA in the proliferative phase coincided with a predominance of perivascular large stellate fibroblasts and their tendency to arrange themselves in an orderly fashion and parallel to the wound surface in AO rats. Somewhat more mature granulation tissue observed at day 5 in AO rats might be the result of coordinated TGF-*β*1-fibroblast response.

More numerous blood vessels in the granulation tissue of DA rats depict better progression of the wound healing process. It might be ascribed to higher expression of VEGF, knowing its role in angiogenesis which maintains a critical role in wound healing. VEGF affects vascular dilation, permeability, endothelial cell migration, and proliferation, which contribute to new vessel sprouting from existing ones as well as new blood vessel formation from endothelial cell progenitors and stabilization of growth of new vessels [51, 75–77]. Less intense wound vasculature in AO rats might be related to their obesity, given reduced blood vessel density and a delay in blood vessel remodeling in diet-induced obesity mice [24] and rats [23], probably as a consequence of severely impaired VEGF expression noted in diabetic and obese mice [78]. The more frequent horizontal arrangement of tubular blood vessels in the lower parts of the wound of AO rats, an indication of the growth of new blood vessels around the wound [79], suggests the effort of the neovascularization process improvement in these rats. More pronounced expression of collagen I in granulation tissue of DA rats also shows a better progression of wound healing in DA rats, as collagen III is replaced by stronger collagen I as wound remodeling progresses [80].

This study showed that the differential rate of wound closure coincided with differential proinflammatory and immunoregulatory cytokine as well as growth factor response in AO and DA rats. Some of these differences seem related to differences in the proneness to obesity. Although this study extended so far known differences in inflammatory/immune responses to a variety of stimuli between two rat strains, it showed, for the first time, immune-based differences in wound healing between rats, which differ in BM and obesity proneness (under ad libitum feeding conditions with normal rodent chow). Our data open the possibility of exploring the relationship between obesity and immune response and vice versa.

## Figures and Tables

**Table 1:** Obesity indices in rats.

	DA	AO
Body mass (g)	226.4 ± 9.8	332.7 ± 18.2*⁣*^*∗∗∗*^
Body length (cm)	21.0 ± 0.4	21.7 ± 0.4
Body mass index (g/cm^2^)	0.52 ± 0.02	0.70 ± 0.03*⁣*^*∗∗∗*^
Lee obesity index (g/cm)	291.1 ± 5.8	318.5 ± 4.5*⁣*^*∗∗∗*^
Food intake (per 100 g b.w)	8.2 ± 1.8	8.6 ± 2.2

*Note:* Results are presented as mean ± S.D. for a total of nine animals per strain. Statistically significant at *⁣*^*∗∗∗*^*p* < 0.001 for AO vs. DA rats.

**Table 2:** Leukocyte count in peripheral blood.

	DA				AO			
	Day 1		Day 3		Day 1		Day 3	
	Without wound	Wounded	Without wound	Wounded	Without wound	Wounded	Without wound	Wounded
Total WBC (×10^9^/l)	8.0 ± 4.7	7.3 ± 3.9	8.8 ± 4.8	9.3 ± 2.6	5.4 ± 2.7	4.9 ± 2.5	5.8 ± 3.4	5.4 ± 1.8
Neutrophils (%)	35.3 ± 3.4	43.9 ± 9.9^#^	42.8 ± 6.4	43.6 ± 9.8	48.0 ± 6.8	34.1 ± 7.4^#^	42.4 ± 12.1	38.8 ± 7.1
Lymphocytes (%)	62.6 ± 2.4	54.0 ± 9.7^#^	59.9 ± 8.4	54.3 ± 9.5	48.7 ± 6.9	63.5 ± 7.3^#^	54.8 ± 12.2	57.8 ± 7.4
Monocytes (%)	0.52 ± 0.27	0.68 ± 0.12	0.76 ± 0.05	0.68 ± 0.19	0.78 ± 0.35	0.62 ± 0.26	0.78 ± 0.13	0.67 ± 0.36
Eosinophils (%)	1.24 ± 0.79	1.10 ± 0.54	0.72 ± 0.42	1.02 ± 0.54	1.33 ± 0.24	1.35 ± 0.30	1.70 ± 0.64	2.35 ± 1.06
Basophils (%)	0.32 ± 0.26	0.27 ± 0.42	0.36 ± 0.17	0.34 ± 0.15	0.35 ± 0.33	0.45 ± 0.25	0.33 ± 0.21	0.33 ± 0.18

*Note:* Results are presented as mean ± S.D. for a total of nine animals per strain. Statistically significant at ^#^*p* < 0.05 for control vs. wounded animals.

Abbreviation: WBC, white blood cell.

**Table 3:** Number and activity of cells migrating into the wound.

Parameters examined	DA	AO
Cell numbers		
Total (×10^6^)	18.9 ± 5.1	20.8 ± 4.2
HIS48^+^ cells (%)	76.1 ± 4.3	38.9 ± 8.5*⁣*^*∗∗*^
CD68^+^ cells (%)	5.2 ± 1.3	9.7 ± 3.7*⁣*^*∗*^
Cell activities		
Intracellular MPO (U/10^6^ cells)	1.47 ± 0.31	0.40 ± 0.03*⁣*^*∗∗*^
DHR^+^ cells (%)	24.4 ± 8.2	23.1 ± 6.3
DHR (MFI)	42.9 ± 1.6	49.2 ± 2.8*⁣*^*∗∗*^
IL-1*β* (pg/ml)	111.1 ± 22.6	170.0 ± 16.3*⁣*^*∗∗*^
IL-6 (pg/ml)	183.1 ± 50.0	132.3 ± 16.2*⁣*^*∗*^
TNF (pg/ml)	203.9 ± 53.3	207.1 ± 82.8
IL-17 (pg/ml)	837.7 ± 173.0	456.2 ± 50.1*⁣*^*∗∗*^
Wound fluid		
IL-1*β* (pg/ml)	193.3 ± 18.8	145.0 ± 26.5*⁣*^*∗∗*^
IL-6 (pg/ml)	2322.8 ± 563.1	2137.5 ± 186.1
TNF (pg/ml)	350.4 ± 83.4	557.5 ± 61.0*⁣*^*∗*^
IL-17 (pg/ml)	895.6 ± 369.9	240.6 ± 167.8*⁣*^*∗∗*^

*Note:* Results are presented as mean ± S.D. for six animals per strain and compared with Student's *t*-test. Statistically significant at *⁣*^*∗*^*p* < 0.05 and *⁣*^*∗∗*^*p* < 0.01 for AO vs. DA rats.

## Data Availability

The data that supports the findings of this study are available from the corresponding author upon reasonable request.

## Nomenclature

AO: Albino Oxford
Arg1: Arginase 1
BM: Body mass
BMI: Body mass index
cDNA: Complementary DNA
DA: Dark Agouti
ECM: Extracellular matrix
EGF: Epidermal growth factor
FGF: Fibroblast growth factor
HE: Hematoxylin and eosin
IGF-1: Insulin-like growth factor-1
IL: Interleukin
iNOS: Inducible nitric oxide synthase
MMLV: Moloney murine leukemia virus
MN: Mononuclear
Mphs: Macrophages
mRNA: Messenger ribonucleic acid
PDGFs: Platelet growth factors
PMN: Polymorphonuclear
RT-PCR: Real-time polymerase chain reaction
TGF-*β*1: Transforming growth factor *β*1
TNF: Tumor necrosis factor
VEGF: Vascular endothelial growth factor.
